# Epidemiological perspective associated with principal risk factors of *Trichinella spiralis* infection in pigs and humans in Egypt

**DOI:** 10.14202/vetworld.2022.1430-1437

**Published:** 2022-06-11

**Authors:** Eman Sayed Mohammed, Asmaa Gahlan Youseef, Asmaa Gaber Mubarak, Amany Sayed Mawas, Fatma Ahmed Khalifa, Wael Felefel

**Affiliations:** 1 Department of Parasitology, Faculty of Veterinary Medicine, South Valley University, Qena 83523, Egypt; 2 Department of Zoonoses, Faculty of Veterinary Medicine, South Valley University, Qena 83523, Egypt; 3 Department of Pathology and Clinical Pathology, Faculty of Veterinary Medicine, South Valley University, Qena 83523, Egypt; 4 Department of Infectious Diseases, Faculty of Veterinary Medicine, South Valley University, Qena 83523, Egypt; 5 Department of Parasitology, Faculty of Veterinary Medicine, Matrouh University, Matrouh 51744, Egypt

**Keywords:** Egypt, enzyme-linked immunosorbent assay, humans, pigs, polymerase chain reaction, *Trichinella spiralis*

## Abstract

**Background and Aim:**

In Egypt, there is a scarcity of recent data on trichinellosis in pigs and humans. Therefore, this study aimed to determine the epidemiological profile and risk factors associated with *Trichinella spiralis* infection as well as to assess the effectiveness of the trichinoscope and digestion technique in diagnosing trichinellosis.

**Materials and Methods:**

Data were collected on 33812 pigs slaughtered during a year at the Al-Basateen abattoir, Cairo Governorate, Egypt. The slaughtered pigs had already been examined by trichinoscope in the abattoir. The diagnostic effectiveness technique was randomly conducted on 170 pork muscle samples, which were examined using the digestion technique. Furthermore, 90 serum samples from high-risk individuals in Qena and Sohag Governorates, Upper Egypt, were analyzed using an enzyme-linked immunosorbent assay.

**Results:**

The investigation revealed that the overall prevalence was 1.06% in pigs by trichinoscope. Of the examined 170 samples, 2.35% and 3.35% were found to harbor *Trichinella* by trichinoscope and artificial digestion, respectively. *Trichinella* was identified as *T. spiralis* using a polymerase chain reaction (PCR) technique. A significant relationship was affirmed between the prevalence of trichinellosis and the sex and age of the examined pigs. Likewise, for the first time, there was a considerable seasonal trend in the prevalence of *Trichinella* with the maximum infection, which was observed during Autumn (1.18%). The prevalence of trichinellosis in humans was 10%, with a significant association with age.

**Conclusion:**

Our findings are intended to serve as a starting point for developing effective preventive and control measures for trichinellosis (as application of Hazard Analysis Critical Control Points (HACCP) in pig farms, stop feeding pigs on garbage as well as, preventing illegal slaughter of pigs outside the slaughterhouses). It also fortifies the establishment of the digestion technique because of its high specificity and sensitivity, although it is difficult to apply to a large number of samples.

## Introduction

Trichinellosis is a worldwide zoonotic parasitic infection of numerous mammals, including pigs and humans with nematodes of genus *Trichinella*, with clinical symptoms which appear mainly in humans. Historically, the biology and epidemiology of the genus *Trichinella* have been intensely associated with swine, which acts as the intermediate host of most concern to humans [1]. Consequently, trichinellosis is considered a disease not only of public health hazard but also represents an economic problem in porcine animal production and food safety. According to the international literature, *Trichinella spiralis* is the species most frequently detected in domestic and wild swine [2, 3], simultaneously it is the most etiological agent responsible for the disease in humans [4].

In pigs, the disease is perpetuated by swill feeding; eating any animal matter such as meat, meat products, and meat by-products especially infected rodents’ carcasses through which the encysted larvae are eaten by pigs and excyst in the small intestine, then mating occurs and the females burrow through the gut wall to give birth to larvae that encyst in the muscles and can live for 10 years causing trichinellosis in humans when consumed [5, 6].

*Trichinella* infection in humans is strongly associated with the consumption of undercooked pork containing larvae. However, a large outbreak of the disease may arise due to the consumption of minced beef illegally mixed with pork of unknown origin or ground in a grinder previously used for contaminated pork [7]. Besides, the severity of clinical symptoms is strongly dependent on the number of infective larvae ingested by the person ranging from asymptomatic to fatality. Severe complications, including myocarditis and encephalitis, may occur remarkably in the elderly or those with an impaired immune system [8].

In the human body, larvae migrate into the intestinal mucosa, then to blood vessels to reach their final location in muscles inducing immunological, pathological, and metabolic disturbances [9]. Immunopathology is characterized by eosinophilia which is responsible for the allergic manifestations typical of trichinellosis. Three cell modification stages occur in acute trichinellosis: Nurse cell, encapsulated larvae, and capillary network surrounding the infected cell [10, 11].

Diagnosis of *Trichinella* infection is crucial to eliminate infected animals from the food chain and avoid the disease in humans. Swine trichinellosis is diagnosed according to the regulatory technique of direct post-mortem trichinoscopy and artificial digestion. Trichinoscopy is rapid and cost-effective, while it is less sensitive in detecting light infection (few number of larvae in muscles) [12]. Artificial digestion is considered more appropriate and flexible, especially in the case of non-encapsulated *Trichinella* larvae [13, 14]. As there are no morphological features to specify larvae, the molecular technique is used for genotyping and confirming the different species of genus *Trichinella* [15].

Trichinellosis is regarded as an emerging disease in some parts of the world, such as South America [16]. Thus, its global importance has motivated the development of several serological methods for the detection of human trichinellosis, such as the indirect fluorescent antibody test, Western blot analysis, Bentonite flocculation, and latex agglutination, enzyme-linked immunosorbent assay (ELISA) is the recommended and most commonly employed approach in human trichinellosis. Compared with other serological devices, ELISA is easy to conduct, sensitive, can be functioned for large-scale testing, and it is the only serological method recommended by the Office International des Epizooties [13, 17].

In Egypt, although several cases of human trichinellosis were reported in different localities such as El-Minya, Assiut, and Sohag governorates, few studies [18, 19] have addressed the issue from a zoonotic point of view. Hence, our study was conducted to evaluate the prevalence of *T. spiralis* infection in Egypt through parasitological, histopathological, molecular, and serological methods and to realize possible risk factors related to *Trichinella* infection among pigs and humans to eradicate the infection from the food chain.

## Materials and Methods

### Ethical approval and Informed consent

This study was approved by the ethical committee of South Valley University, Qena, Egypt (No. 14/24.5.2021). Also, Oral consent was obtained from each participant.

### Study period and location

The study was conducted from January to December 2020. Pigs study was executed in Al-Basateen abattoir, Cairo Governorate, Egypt Google map (31.2064775, 29.9260792).

### Study design and sampling

Data about 33812 slaughtered pigs were collected through a standard form of abattoir archives, including pigs’ sex, age, weight, and farming source. Al-Basateen abattoir is currently the only one authorized to slaughter pigs and distribute them to all governorates as one of the precautionary measures taken by the Egyptian authorities to confront the swine flu since 2009. One hundred and seventy muscle samples (diaphragm and skeletal muscles including biceps femoris and gluteus medius) were collected separately in labeled plastic bags and stored at 4°C for diagnostic accuracy test of digestion. The samples were also examined through histopathological and molecular methods.

The human survey was conducted on 90 serum samples of high-risk individuals who have a case history of eating pork and pork products in Qena and Sohag Governorates (southern part of Egypt), where pigs’ meat was distributed. A venous blood sample of 5 mL was collected from each participant and allowed to clot and centrifuged at 1000 g for 5 min. Sera were stored at −20°C until examined through ELISA. The questionnaire included sex and age.

### Trichinoscope method

A total of 33812 slaughtered pigs were examined in the abattoir via this method through which one gram of meat was cut into small pieces and squeezed between two compressorium glass plates, which were firmly messed up together, resulting in a thin, transparent layer of meat that is examined under a binocular microscope (20×) [12].

### Digestion technique

Four grams of each 170 examined muscle sample was digested in 100 mL of artificial digestive fluid containing 1% pepsin (1:10.000; U.S. National Formulary) and 1% hydrochloric acid. An electric stirrer was used to agitate the mixture for 3 h at 37°C continuously. Excess supernatant was expelled, and sediment was sieved and transferred to a Petri dish for larvae inspection under a dissecting microscope (20×) [14].

### Molecular identification

Deoxyribonucleic acid (DNA) extraction: A pool of the recovered *Trichinella* larvae from six positive samples was submitted to the DNA extraction for molecular identification. DNA extraction was achieved using the QIAamp DNA Mini kit (Qiagen, Germany, GmbH) under the manufacturer’s instructions and stored at −20°C for further use.

Polymerase chain reaction (PCR) amplification: Oligonucleotide primer (5’-GCAGCTATGGATGTTCAGGTG-3’, and 5’-TACGGCTGACAGCATGATT TG-3’) was used for the detection of 109 bp of TsCP glyceraldehyde-3-phosphate dehydrogenase (GAPDH) gene (Metabion, Germany) [20]. The PCR was conducted in an Applied Biosystem 2720 thermal cycler program as follows: denaturation at 94°C for 5 min followed by 35 cycles of 94°C for 30 s, annealing temperature was 60°C for 30 s and extension at 72°C for 30 s and final extension at 72°C for 7 min.

PCR product analysis: The scale of the amplicons was determined using a gene marker 100 bp ladder (Fermentas, Germany). The result was recorded using a gel documentation framework (Alpha Innotech, Biometra, Germany).

### Histopathological examination

Four muscle samples from infected pigs obtained at necropsy were examined after compression between two glass slides, and free non encapsulated larvae were observed. Then muscle tissue samples were fixed in 10% neutral buffered formalin solution (pH 7.4), passed in ascending grades of ethyl alcohols, embedded in paraffin, then cut (5 μm) and stained with hematoxylin and eosin for visualization of the general tissue morphology including muscle degeneration, inflammatory reaction and encysted larva of *Trichinella* in muscle tissue. The slides were examined using a light microscope (Olympus BX51, Tokyo, Japan) with a camera (Olympus E-182 330, Olympus Optical Co., Ltd., Japan). Five slides were examined for each block [21].

### ELISA

Ninety human serum samples were screened for the presence of anti-*Trichinella* *spiralis* Immunoglobulin (Ig)G antibodies by a commercial indirect ELISA kit (NovaTech GmbH, TRIG0480, Germany) as instructed by the manufacturer. According to the manufacturer’s recommendation, results were reported in Nova-Tech Units (NTU). Sera with values of <9 NTU were considered negative; those between 9 and 11 NTU were considered equivocal and were repeated with a fresh sample within 2–4 weeks. If the result is equivocal again, the sample was judged as negative, and those with values >11.0 NTU were considered positive for *T. spiralis* IgG antibodies.

### Statistical analysis

Statistical analyses were performed using the Statistical Package for the Social Sciences Statistics software, version 24. Pearson’s Chi-square (p-value at 5%), Fisher’s exact test and Monte Carlo test were employed to predict the association between qualitative categorized variables. Odds ratio and binary logistic regression were used to predict the risk factors. Receiver-operating characteristic curve value as a diagnostic accuracy-test was executed to estimate the efficiency of digestion technique as a screen test versus trichinoscope.

## Results

### Pigs’ study survey

Data recorded for a 1-year screening of trichinellosis in Al-Basateen abattoir revealed that the overall prevalence of *T. spiralis* was 1.06% among 33812 slaughtered pigs using trichinoscope. Concerning the associated sex risk factor, the current study revealed a significant relationship (χ^2^ = 260.22, p = 0.0001*) between the sex of slaughtered pigs and trichinellosis as a higher prevalence was observed in females (80.77%) when compared to males (19.22%). The age-wise analysis exposed a higher infection rate in the age group > 1 year (52.92%) as compared to ages <1 year (47.07%) with a statistically significant difference (χ^2^ = 200.93, p = 0.0001*). In contrast, no significant relationship could be detected between trichinellosis and slaughtered pigs’ bodyweight, even though pigs weighing > 50 kg showed a higher incidence of *Trichinella* (66.85%) (Table-1). Seasonally, the highest prevalence rate of trichinellosis was during Autumn (1.18%), followed by Summer (1.17%) and Winter (1.08%), while Spring exhibited the lowest rate (0.84%), as described in Table-2.

**Table 1:** The prevalence of *Trichinella spiralis* among slaughtered pigs relative to sex, age, and body weight by trichinoscope.

Number of slaughtered pigs	Infected pigs	χ^2^	p-value	Odd ratio

	No. (%)			
33812	359 (1.06)			
Sex		260.22	0.0001*	0.15 (0.12–0.2)
Females (No. = 13322)	290 (80.77)			
Males (No. = 20490)	69 (19.22)			
Age		200.93	0.0001*	4.05 (3.29–5)
<1 year (No. = 6188)	169 (47.07)			
>1 year (No. = 27624)	190 (52.92)			
Body weight		0.01	0.9203	1.01 (0.81–1.26)
<50 kg (No. = 11124)	119 (33.14)			
>50 kg (No. = 22688)	240 (66.85)			

* Significant value

**Table 2:** Seasonal prevalence of *Trichinella spiralis* in slaughtered pigs by trichinoscope.

Season	No. of the slaughtered pig	Infected case No. (%)	χ^2^/p-value	Odd ratio
Winter	7052	76 (1.08)		1.02 (0.79–1.3)
Spring	9545	80 (0.84)	6.69	0.79 (0.62–1.01)
Summer	9039	106 (1.17)		1.1 (0.89–1.37)
Autumn	8176	97 (1.18)	0.0825*	1.12 (0.89–1.4)
Total	33812	359 (1.06)		

* Significant value

**Figure-1:**
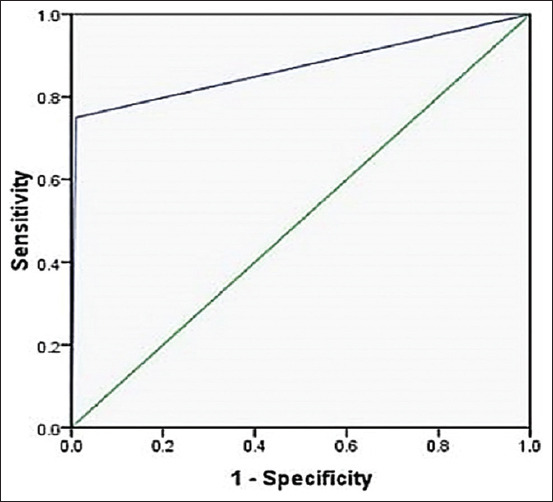
Receiver-operating characteristic curve.

Through checking the diagnostic efficacy of the digestion technique, out of 170 muscle specimens, 4 (2.35%) and 6 (3.35%) were found to harbor *Trichinella* larvae using trichinoscope and artificial digestion, respectively (Table-3). Compatibly, the sensitivity and specificity of the diagnostic accuracy of artificial digestion technique compared to trichinoscope (a gold standard [reference method] of *Trichinella* infection diagnosis) was determined as 100% and 98.7%, respectively, with area under the curve (0.994) (0.983–1.005) and p (0.001) value as illustrated in Table-4 and Figure-1.

**Table 3:** The prevalence of *Trichinella spiralis* by trichinoscope and artificial digestion methods.

No. of Pig samples	Infected cases		Statistical significance	Odd ratio Logistic regression	Significance

	Trichinoscope No. (%)	Artificial digestion No. (%)			
170	4 (2.35)	6 (3.35)	0.301^	4.781 (0.487–46.963)	0.083

^ = Fisher’s Exact Test

**Table 4:** The diagnostic accuracy test of artificial digestion.

Variables	Percentage
Diagnostic efficiency	98.8
Sensitivity (95% CI)	100 (39.5–100)
Specificity (95% CI)	98.7 (95.2–99.7)
PPV (95% CI)	66.6 (24.1–94.0)
NPV (95% CI)	100 (97.1–100)
PLR (95% CI)	83.3 (20.9–329.1)
NLR (95% CI)	0 (0–NaN)
DA	66.6
Prevalence (95% CI)	2.35 (0. 75–6.29)
AUC	0.994 (0.983–1.005)
p-value	0.001

PPV = Positive predictive value, NPV = Negative predictive value, PLR = likelihood ratio for positive results, NLR = likelihood ratio for negative results, DA = Discrimination ability (PPV+NPV-100) 100%, AUC = Area under the curve.

### Molecular analysis

**Figure-2:**
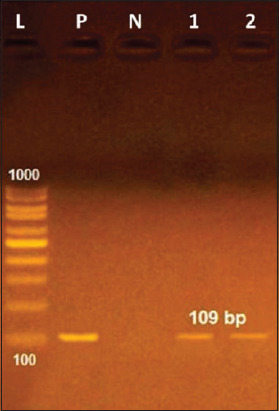
PCR analysis of the recovered *Trichinella* from slaughtered pigs, L=Ladder, P=positive control, N=Negative control, lanes 1, 2=PCR product of smaller size band of 109 bp *GAPDH* gene for characterization of *Trichinella spiralis*. PCR=Polymerase chain reaction.

PCR-based assay resulted in a distinctly smaller size band of 109 bp in PCR of *GAPDH* gene as displayed in Figure-2.

### Histopathology of muscle tissue

**Figure-3:**
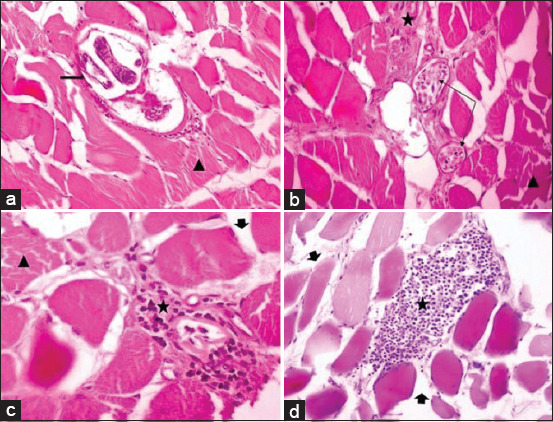
Cross-sections of muscle biopsy of pigs showing: encysted trichinella larvae in a transforming muscle cell with minimal inflammation (thick arrow), hyaline eosinophilic degeneration of muscle fibers and loss of its cross striations (triangle), multiple encysted trichinella larvae within degenerated muscles (thin arrows), edema (short thick arrow) and mononuclear inflammatory reaction between degenerated muscle mainly eosinophil, macrophages and some are polymorphic cell infiltrate surround trichinella larvae (stars). a and c: 400×, b and d: 200×, hematoxylin and eosin stain. Scale bar = a and c: 50 μm, b and d: 100 μm

Muscle tissue revealed encysted *Trichinella* larvae surrounded in some cases with a minimal inflammatory reaction, and other cases have focal inflammatory infiltration (macrophages, eosinophils, and polymorphonuclear cells). These inflammatory infiltrations were seen around the migrating larvae, between the degenerated muscles and un-parasitized muscle fibers. Diffuse hyaline eosinophilic degenerative changes all over the muscle bundles and edema in-between were also observed (Figure-3).

### Human serological survey

A total of 90 at-risk persons (51 males and 39 females) aged 25-65 years with a mean age of 48.11 ± 9.289 were eligible to participate in this survey. Based on ELISA, the seroprevalence rate of *T. spiralis* (IgG) in humans was 10% (9/90) distributed as 5.56% (2/36) and 12.96% (7/54) in Qena and Sohag Governorates, correspondingly. The incidence of trichinellosis among males was higher (13.73%) than females (5.13%), with no significant sex difference. Results of the statistical analysis disclosed that anti-*Trichinella* seropositivity was associated with age (p = 0.263); according to the results, acquisition in group 45 < 55 years was 21.05%, followed by the age group 55 < 65 (11.76%). While, among the 25 < 35 years group, it was 5%, at the time, the infection could not be detected in the 35 < 45 age group (Table-5).

**Table 5:** Seroprevalence of *Trichinella spiralis* in humans stratified by location, sex, and age.

Variable	Infected cases	Statistical significance	Odd ratio	Significance

	No. (%)			
Location				
Qena Governorate (No. = 36)	2 (5.56)	0.306^	0.395 (0.077–2.020)	0.224
Sohage Governorate (No. = 54)	7 (12.96)			
Total (90)	9 (10)			
Patient sex				
Male (No. = 51)	7 (13.73)	0.290^	2.943 (0.576–15.039)	0.167
Female (No. = 39)	2 (5.13)			
Patient age (years)				
25<35 (No. = 20)	1 (5)	b = 3.817 P*=* 0.263	4.554 (9.443–2.196E10)	0.018
35<45 (No. = 17)	0 (0)		1.25E+12	0.997
45<55 (No. = 19)	4 (21.05)		155.408 (1.981–1.219E4)	0.023
55<65 (No. = 34)	4 (11.76)		References	0.03
Minimum = 29 years			1.587 (1.067–2.360)	0.023
Maximum = 65 years				
Mean±SD 48.11±9.289				

^b^ Monte Carlo test, ^Fisher’s exact test

## Discussion

Several outbreaks of trichinellosis were reported in many parts of the world, with varying levels of incidence depending on the country’s cultural food preferences, such as Belgium in 2014, and France and Serbia in 2017 [22, 23]. In Egypt and surrounding countries, sporadic cases occur as they are Muslim-majority countries that refrain from eating pork and pork products. During this study, data collected from the abattoir over the year revealed that the overall prevalence of *T. spiralis* was 1.06% among 33812 slaughtered pigs screened with trichinoscope for a year. This lower incidence of *Trichinella* infection was almost identical to other studies conducted by Morsy *et al*. [24] and Dyab *et al*. [12] in Egypt and recorded incidences of trichinellosis at 1.69% and 1.08%, respectively. This result is consistent with the fact that the prevalence rate of trichinellosis in pigs has dropped in recent years, which might be due to the restrictions developed by the authorities to raise swine since 2009 after slaughtering thousands of pigs to prevent swine flu spreading, and what followed in dramatic change in feeding habits for pigs from garbage to wedding food remains. However, the risk of infection can still be considered high because hundreds of infected pigs correspond to this percentage.

The data showed that trichinellosis among pigs varied significantly according to sex, from 80.77% in females to 19.22% in males. A similar study recorded a lower rate in males (12.6%) than females (14.1%) [25], which could be explained by the feeding behavior of female pigs, who are known to be more voracious in their feeding habits, especially during mating and pregnancy, than males, who are known to be more selective. At the same time, disagreed results were detected by Sayed *et al*. [19] and Konwar *et al*. [26] as 4.8, 2.2, and 3.38, 1.96% in male and female pigs, respectively.

The obtained results exhibited a significantly higher incidence of trichinellosis in pigs in the age group >1 year (52.92%). Ojodale *et al*. [25] also found that infection in adult pigs exceeds that of juvenile pigs (14.6 and 10.7%, respectively). Several authors reported incompatible results [19, 26]. It is practically possible that older pigs with outdoor access are at greater risk of trichinellosis due to gain access to garbage dumps and eating the dead rodent’s carcasses.

Concerning body weight, an insignificant difference could be detected between trichinellosis and slaughtered pigs’ weight in this study. However, the higher incidence of *Trichinella* infection was determined in pigs weighing more than 50 kg (66.85%). Díaz *et al*. [27] also observed no statistically significant effect of weight on the prevalence of trichinellosis in pigs. This may be due to the rising demand for food and so increasing the risk of infection with higher body weight.

In Egypt, our study was the first to demonstrate a significant relationship between the seasonality and prevalence of *T. spiralis* in pigs. Autumn showed the highest prevalence rate (1.18%), while Spring showed the lowest rate (0.84%). This is opposite to a study conducted in Spain [27], during which the seasonal prevalence of *Trichinella* in wild boars was the highest during Summer (0.33%), followed by Winter (0.30%) and Autumn (0.29%), while no cases were detected in Spring. This discrepancy in the prevalence is possibly due to differences in climatic conditions, host, and nature of food. Moreover, the consumption of pork in Egypt is prohibited for Muslims, so the number of slaughtered pigs increases during the Christian holiday season in autumn, which may be a reason for the increased spread of infection during this season.

Diagnosis of *T. spiralis* by digestion technique (3.35%) showed more sensitivity than trichinoscope (2.35%) examination, which is still used in many countries by which each pig is examined individually, and the results can be obtained after slaughtering directly. However, it is less reliable in very mild infections and very difficult to determine the non-encysted larvae. Therefore, the muscle digestion technique is recommended due to its elevated sensitivity.

The result we obtained through artificial digestion was similar to that recorded by Sayed *et al*. [19] (4%) but not the same as Hassanain *et al*. [18], who recorded a prevalence rate of 16%. At the same time, other studies registered a lower prevalence of swine trichinellosis [26, 28] at 2.1% and 2.87%, respectively. Conversely, using different techniques, Abdel-Hafeez *et al*. [29] could not detect *T. spiralis* larvae in slaughtered pigs in El-Minia Governorate. This conflict in the prevalence rate may be due to differences in the measures for detection and control, management, and feeding of pigs in different countries and hygienic conditions for rearing pigs on farms. Moreover, *T. spiralis* was the main species identified in our study through molecular technique, similar to the previous studies [19, 30].

Regarding the histopathological examination, muscular sections infected with *T. spiralis* revealed myositis, which was defined by inflammatory cell reaction, mainly eosinophils. Hyaline eosinophilic muscular degeneration and edema were also observed. The principal pathology in the acute phase of trichinellosis starts with the release of histaminase enzyme, which is responsible for eosinophilia (type I hypersensitivity reaction), followed by increased levels of mast cells, eosinophils, and parasite-specific IgE production, leading to inducing *Trichinella* damage to the blood vessels which caused by eosinophil cationic protein, and eosinophil peroxidase [9]. Migration of *Trichinella* larvae in the muscle is associated with inflammatory and allergic reactions stimulating eosinophil infiltration [31]. Infected muscle cells in *T. spiralis* transformed into nurse cells, followed by cyst formation with surrounded local myositis [11]. After cyst formation, focal to absent inflammatory reaction appears as a host immune response consisting of eosinophils, neutrophils, lymphocytes, and encysted larvae to replace muscle fiber. The capsule surrounding *T. spiralis* larvae is considered its own home in the degenerated muscle and is composed of collagenous wall and cellular components, which is named nurse cell due to its protective function against the parasite [31].

*Trichinella* infection constitutes a risk for human health, along with causing considerable economic losses in world food production. It has been estimated that about 10000 cases of human trichinellosis occur globally each year, with an approximate mortality rate of up to 0.2% [32].

The seroprevalence rate of *T. spiralis* infection in humans tested by ELISA was 10% (9/90). Other studies which were conducted in El-Minya governorate, Egypt and Northern Laos recorded higher rates of human trichinellosis [18, 28, 32] at 56.0%, 67.6%, and 19.1%, respectively. While in China lower results have been recorded [33, 34] as 3.57% and 3.19%, respectively. These results may be attributed to the general ignorance of the public about trichinellosis, its source of infection, symptoms, and the sequelae of the disease. In addition, most clinicians lack the experience in diagnosing as it can be confused with other febrile infections and myositis. Moreover, illegal slaughtering of pigs (in other places out of slaughterhouses) without any hygienic measures or veterinary supervision plays a key role in transmitting trichinellosis either to humans or animals.

In our study, the prevalence of seropositivity was higher in Sohag (12.96%) than in Qena Governorate (5.56%). The location can be one of the risk factors for *Trichinella* infection, as confirmed by Söderberg *et al*. [35]. The places that suffer from a high poverty rate are more susceptible due to the dependence on home-prepared traditional pork products for a larger proportion of their diet [5], accompanied by people’s attitudes.

Similarly, males were more vulnerable to infection (13.73%) than females (5.13%). This was coordinated with the findings reported by several researchers [28, 36, 37]. Contrary to the achieved results, several authors recorded a higher prevalence of trichinellosis in females than males [19, 35]. These differences may be because samples in this study were taken from more men who raised pigs. In our study, location and sex risk factors associated with *T. spiralis* seropositivity were not statistically significant.

In contrast, a significant correlation between *T. spiralis* seroprevalence and patients’ age was reported in the present study that the highest rate of *Trichinella* infection was recorded in the age group of 45<55 years (21.05%) followed by 55<65 years old group (11.76%). Similar findings were obtained by other studies [28, 36, 38], which revealed that the seroprevalence of *Trichinella* antibodies increased by age. On the other hand, Addo *et al*. [39] found no variance in the positivity according to age.

## Conclusion

This work presented a comprehensive study of several diagnostic techniques to screen *Trichinella* in pigs and humans in Egypt, focusing on risk factors. A significant correlation between swine trichinellosis, sex, and age of pigs was concluded. The diagnostic accuracy test revealed that the digestion technique is more precise than using a trichinoscope, but its application to a large number of samples may be difficult, so in our study, the samples number examined by this technique was limited. On the other hand, the risk of *Trichinella* antibody detection in humans was associated with age besides specific food practices. Therefore, prevention efforts could be targeted toward meat safety surveillance in abattoirs to prevent human infection by prohibiting the feeding of raw swill to pigs and ensuring adequate farm biosecurity such as vermin control meat inspection methods for *Trichinella* detection. Future research in different areas of Egypt will be useful to highlight the epidemics of trichinellosis to determine the potential public health concern.

## Authors’ Contributions

ESM, AGY, and AGM: Conceived the idea and study design. WF: Sample collection and data analysis. ESM, AGY, AGM, FAK, and ASM: Parasitological and histopathological examination. ESM, AGY, AGM, and ASM: Drafted, reviewed, and edited the manuscript. All authors have read and approved the final manuscript.
